# Clinical Outcome of Carotid Body Paraganglioma Management: A Review of 10-Year Experience

**DOI:** 10.1155/2020/6081273

**Published:** 2020-07-20

**Authors:** Ahmed Elsayed Fathalla, Mohammad Ahmad Elalfy

**Affiliations:** National Cancer Institute, Cairo University, Giza, Egypt

## Abstract

**Background:**

Carotid body paragangliomas are rare neoplasms usually benign, however sometimes presenting as highly aggressive tumors. Surgery is the main line of treatment.

**Purpose:**

To study and describe clinical presentations, surgical approaches, postoperative complications, and treatment outcomes.

**Materials and Methods:**

A single-institution retrospective analysis of 19 cases with carotid body paragangliomas who were candidates for surgery from January 2009 through January 2019 with a mean follow-up period of 58.8 months.

**Results:**

The mean age was 46 years with the female predominance of 63%. The mean size of the tumor was 4.3 cm. All cases were presented with a painless pulsating neck lump located anteriorly at the level of the hyoid bone. Neck US was done in all cases as a primary screening investigation. CT scanning was the second main investigation performed in 17 cases (89.5%) revealing tumors attached to the carotid artery at its bifurcation. Urinary catecholamine metabolites were measured in all cases to rule out familial functioning types. 5 cases (26.3%) were malignant. All cases were surgically approached through transcervical transverse incision. 11, 5, and 3 cases were classified as Shamblin's type II, III, and I, respectively. All tumors were R0 resected with nodal neck dissection conducted in the malignant group. Major complications occurred in 4 cases (21%) during tumor dissection from the adventitia of carotid bifurcation. ECA ligation was performed in one case (5.3%). 2 patients (10.5%) suffered XII nerve paralysis. Carotid artery blowout occurred in one patient (5.3%) and was immediately controlled. No operative mortality occurred. All patients were free of disease during the follow-up period. 4 malignant cases (21%) suffered a systemic relapse to bone and lung metastasis justifying adjuvant chemotherapy, radiotherapy, or both.

**Conclusions:**

Surgery is the treatment of choice for carotid body paragangliomas. Complete R0 resection should be justified especially in case of malignancy. Adjuvant chemotherapy or radiotherapy is an option for patients with primary malignancy or relapse.

## 1. Introduction

Paragangliomas (PGs) are paraganglionic tumors of chemoreceptor cell origin. This term “paraganglia” was first described by Kohn in the early 20^th^ century and is the most appropriate nomenclature from the embryologic aspect [1].

They are distributed paraxially in the trunk mainly related to major arteries and cranial nerves of the pharyngeal arches origin. WHO classification has designated paragangliomas by location (i.e., carotid, vagal, jugular, laryngeal, and tympanic paragangliomas) [2].

Carotid body paragangliomas (CBPs) are also known as chemodectomas because of the physiological function of the carotid body as a chemoreceptor. “Nonchromaffin” refers to the histologic staining which distinguishes these paragangliomas from the chromaffin-reacting tissue of the adrenal medulla [3].

CBPs are rare highly vascular tumors with an incidence of only 0.3% of all paragangliomas and 60% of head and neck paragangliomas, followed by jugulotympanic and vagal PGs [4].

CBPs are more prevalent in middle-aged females and typically present as slowly growing mass. They can remain asymptomatic for years. Clinically, the mass is typically vertically fixed to the carotid bifurcation (Fontaine sign) [5].

10% of CBPs may present with cranial nerve palsy (IX, X, recurrent laryngeal, and XI), involving the sympathetic chain causing Horner syndrome. Typically, they are solitary but can be multicentric, particularly in familial syndromes (Carney's syndrome and MEN types II A and B) which constitute 28% of CBPs [6].

Most patients do not have functioning CBPs. However, symptomatic patients with headaches, excessive sweating, and palpitations should be evaluated with 24 h urine collection for catecholamine metabolites (metanephrine and VMA) as well as serum catecholamines. This is an important recommendation for proper anesthetic safety and appropriate alpha- and beta-blockade [7].

Shamblin described three different stages of CBPs. Type I consists of a small tumor easily dissected from the adjacent vessels in a periadventitial plane. Type II tumors are larger and partially surround the vessel. Type III tumors are large and completely surround the carotid bifurcation [8]^.^

Fine-needle aspiration (FNA) of CBPs is generally not recommended as it may result in significant hemorrhage; however, aspirations in unsuspected cases may show moderate cellularity of small groups arranged in a “pseudorosette” pattern [9].

The majority of CBPs carry a benign behavior and only 6% are malignant. Malignancy is defined mainly by a spread to lymph nodes (LNs) or distant metastases. The reported 5-year survival rate based on the National Cancer Database is about 60% when regional LN metastases were found [10].

Several radiological investigations were identified to evaluate CBPs. Contrast-enhanced CT scans demonstrate an enhancing mass at the carotid bifurcation and detail any fine osseous changes. Contrast-enhanced MRI also shows a hyperintense T2-weighted image. Due to their vascularity, CBPs have a “salt-and-pepper” pattern on MRI caused by high-velocity flow voids (black dots) and foci of hemorrhage or slow flow (white dots). Angiography is sometimes used for patients undergoing resection demonstrating splaying of the internal and external carotid arteries “Lyre sign” [11–13].

^123^I-MIBG scans aid in localization, especially in occult or familial types. ^111^In octreotide (a somatostatin analog) may also be sensitive. Positron emission tomography (PET) using ^18^fluorodeoxyglucose (^18^FDG-PET) shows avid uptake by the tumor cells [14, 15].

Conventional therapy for CBPs is surgical resection. The basic principle of surgery involves locating and preserving the cranial nerves prior to dissecting the tumor as the XI nerve, ansa cervicalis, X nerve, and superior laryngeal nerves are often embedded within the capsule of the tumor. Large tumors may involve the sympathetic chain and IX nerve as well [16].

Radiotherapy (RT) or chemotherapeutics (CTH) theoretically may be successful in ceasing the growth of CBPs, but complete regression with these modalities alone is rare. Thus, it is indicated in large unrespectable and recurrent tumors [17].

## 2. Materials and Methods

Retrospective analysis of all cases presented at NCI-Cairo University with H&N CBP candidates for surgery from January 2009 to January 2019 was approved by the ethical committee. 19 cases were included. Data collected from patients archive at the statistical department included demographic features (age and sex), tumor characteristics (type, grade, stage, primary, or recurrent), type of surgery and sequelae (loss of nerve function), treatment (CTH, RT, or both), and outcome. Failure patterns were classified as local recurrence and distant metastasis.

## 3. Results

Due to the rarity of CBPs, only 19 cases were included. The best methodology to describe such rare tumors is “Descriptive Epidemiology” which evaluates separately age, sex, size of the tumor, presenting symptoms, different managements, complication, and outcomes.

### 3.1. Age and Tumor Size

Patients' age ranged from 35 to 57 years with a mean age of 46 years. 7 cases were males (36.8%) and 12 cases were females (63.2%) with a male-to-female ratio of 1 : 1.7. The size of the tumor ranged from 3 to 10 cm with a mean size of 5.4 ± 2.1 cm. 13 cases (68.4%) were right-sided; however, this peculiar side does not seem to carry any significance as does bilaterality which is seen in the familial types.

### 3.2. Symptomatology

The most common presentation was a painless pulsating neck lump located anteriorly to the sternomastoid at the level of the hyoid bone. The lumps were horizontally mobile but not vertically with palpable thrill and bruit heard in all cases. They progressed slowly over a period of 7–12 months except 3 patients for whom there was a progressive neck lump for more than 5 years. Patient complaints are shown in Table 1.

### 3.3. Investigations

**Figure 1:**
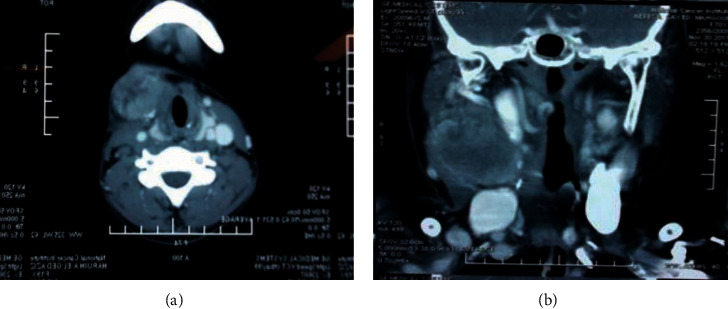
CT scan (axial and coronal views) for 2 patients showing right-sided neck mass diagnosed later as benign CBT.

The most common investigation in our study was neck US carried out in all cases as primary screening (19 cases, 100%). Contrast-enhanced CT was performed in 17 cases (89.5%) and showed tumors attached to the carotid artery at its bifurcation as shown in Figure1. Contrast-enhanced MRI was needed in 9 cases (47.4%). Preoperative radio-guided embolization (either US or CT) was not needed in any of our cases. This latter technique is sometimes needed for large tumors in order to decrease the incidence of intraoperative bleeding, although it might be associated with an increased risk of transient ischemic attacks and ischemic strokes. We concluded that there was no familial pattern, especially in the malignant cases, in light of negative family history and absence of detection of bilateral cases. Urinary catecholamine metabolites were also evaluated in all 19 cases (100%) and were within normal limits. Contrast-enhanced CT abdomen was done for all 19 patients (100%) and was normal.

### 3.4. Treatment

**Figure 2:**
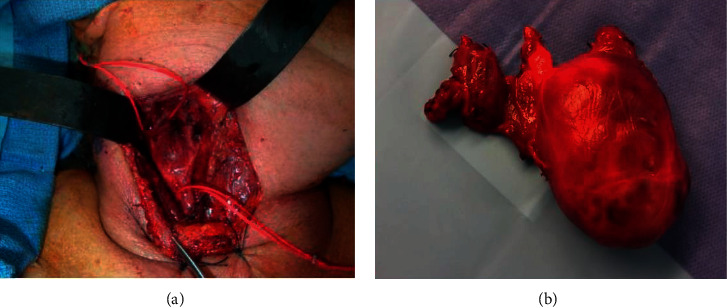
Intraoperative view of a case with malignant CBT (a) showing splaying of the carotid bifurcation. Postoperative specimen of the same patient (b) with an attached metastatic lymph node.

All 19 patients were candidates for surgery. Tumors were approached transcervically through transverse incision. During surgery, 11 cases (57.9%) were classified as Shamblin's type II, 5 cases (26.3%) as Shamblin's type III, and 3 cases (15.8%) as Shamblin's type I. Although they varied in size, all tumors were completely R0 resected. Neck dissection was conducted in 5 cases (26.3%) with malignant CBP confirmed during surgery through frozen section analysis done to the suspected LNs as shown in Figure 2.

### 3.5. Operative Technique

After induction of endotracheal intubation under general anesthesia, patients were placed in the supine position with head extended and rotated to the other side of the tumor. We used a transcervical approach by performing the incision through the upper neck crease. The upper and lower subplatysmal skin flaps were elevated with meticulous dissection using electrocauterization to avoid significant blood loss. The fascia on the anterior border of the sternomastoid was incised, and the muscle was retracted laterally to expose the carotid sheath proximal and distal to the tumor. Several hyperplastic nodes were often encountered with the mass.

We always sent one of the nodes for frozen examination to detect any malignant changes. The carotid sheath was incised in the lower part of the neck to expose the common carotid artery, then dissected circumferentially, and secured with vessel loops. Further identification and isolation of internal jugular vein and vagus, spinal accessory, and hypoglossal nerves were performed. Meticulous dissection all around the tumor, including the embedded vessels, was achieved by bipolar cauterization in order to isolate the external and internal carotid arteries with vessel loops. We then started to dissect along the common carotid to expose the bifurcation. At this point, the carotid artery and its bifurcation were either partially embedded, with an indentation in the tumor rather than circumferential encasement (Shamblin type II tumor), or the tumor surrounded the common carotid artery and its bifurcation and the tumor needed to be incised and bisected to dissect out the artery (Shamblin type III tumor). We first performed dissection of the internal carotid artery to protect it from injury as nearly all the blood supply to this tumor is derived from the branches of the external carotid artery. We performed dissection of the tumor using two approaches: either meticulous peeling with electrocauterization with ligation of the feeding vessels or dissection through the subadventitial plane. In case of bleeding, we applied vascular clamps proximally and distally and kept the systolic blood pressure high to allow administration of intravenous anticoagulants and minimize intracranial hypoxia.

### 3.6. Complications

ECA was ligated in 1 patient (5.3%) as a result of its iatrogenic injury to control bleeding with no postoperative sequelae. 2 patients (10.5%) with Shamblin type II tumors suffered XII nerve paralysis as a result of resection of an involved nerve segment within the tumor. Carotid blowout occurred in 1 patient (5.3%) with XII nerve injury on day 2 postsurgery from the site of the resected part of the common carotid artery at its bifurcation. It was immediately controlled by suturing the defect with no later catastrophic sequelae. No internal carotid artery reconstruction was needed.

### 3.7. Follow-Up

Of the 5 malignant CBP cases, 4 of them suffered a systemic relapse to either bone only, lung only, or both. Adjuvant therapy was given accordingly either CTH alone, RT alone, or concomitant RT/CTH (Table 2).

**Figure 3:**
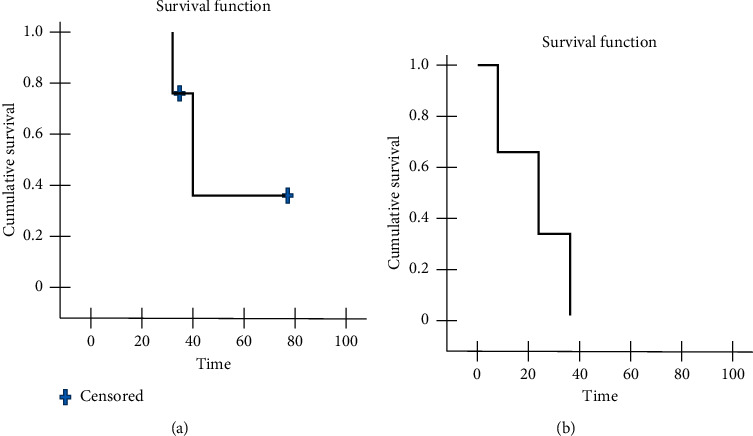
Overall survival (a) and disease-free survival (b) for malignant CBT cases.

In cases of relapse, CTH was given either alone or in combination with RT in the form of Adriamycin-ifosfamide regimen as for other soft tissue sarcomas protocols. In our cohort, we observed 4 cases (18%) with distant metastases to lung , 2 of them showed further metastases to bone. They received both CTH and palliative RT to the bone. Conventional RT was given only as palliative care in case of bone metastasis. Their overall survival and disease-free survival are displayed in Figure 3.

## 4. Discussion

CBPs are rare paraganglionic tissue tumors of neuroendocrine origin. Due to this origin, many histopathologic stains used to identify these tumors (i.e., chromogranin, synaptophysin, serotonin, and neuron-specific enolase) can be positive for other neural-crest tumors, a limitation which should be considered in their differential diagnosis [18].

They are usually present between the 5^th^ and 7^th^ decades of life as a lateral neck mass. The most common presentation in our cohort was painless pulsating neck lump located anteriorly to the sternomastoid at the level of the hyoid bone with horizontal mobility and palpable thrill in all 19 cases (100%) that progressed slowly over the years.

The most important step in resection is superior and inferior control of the blood vessels. This includes identification of the internal jugular vein, common and internal carotid arteries, and placing vessel loops on each. It is difficult to ascertain whether the carotid will need to be replaced with a reverse saphenous vein graft until the intraoperative dissection of the tumor occurs. If there is no good separation between the tumor and artery, then 360° dissections of the tumor and artery are performed. The vascular surgeon then performs a vein graft of the internal carotid [19].

Injury to the carotid vessels will require clamping of the common or internal carotid arteries plus temporary heparinization followed by vascular reconstruction. 65% of patients who had their internal carotid artery ligated had a stroke and 25% of patients who passed their balloon occlusion test had a delayed stroke [20].

In this cohort, ECA was ligated in only 1 patient (5.3%) due to ECA injury to control bleeding with no postoperative sequelae. Carotid blowout occurred in another patient (5.3%) on day 2 postoperatively from the site of the resected part of the CCA at its bifurcation and was immediately sutured with no sequelae. Careful subadventitial dissection and control of the proximal and distal carotids minimizes vascular complications. The size of the tumor and involvement of the carotid artery predict vascular complication risks. The highest risk is observed in tumors >5 cm and/or Shamblin type III. Patients with large tumors also have more frequent postoperative cranial nerve injuries [21]. In a recent larger study, Kim et al. emphasized the value of preoperative detection of tumor dimensions and distance from the base of the skull in combination with the Shamblin grade to better predict bleeding and cranial nerve injury risk. They report that every 1 cm decrease in the distance to the skull base results in a 1.8-fold increase in >250 mL of blood loss and a 1.5-fold increase in the risk of cranial nerve injury. By correlating these variables together, they found that both Shamblin grade and distance from the skull base were correlated with bleeding and cranial nerve injuries; however, tumor volume was correlated only with bleeding [22].

Usually, the most commonly injured nerve is the superior laryngeal nerve which supplies the cricothyroid muscle and provides sensation to the supraglottic larynx resulting in some degree of aspiration and voice changes (inability to create high-pitched sounds). The X nerve itself may be also injured leading to vocal cord paralysis with resultant hoarseness and aspiration. When combined with a superior laryngeal nerve paralysis, as in cases with a high vagal injury, aspiration is significant because the larynx is now totally anesthetic. This may be compensated by the contralateral vocal cord over time; however, if the problem persists, vocal cord medialization procedures should be performed [23].

Speech and swallowing problems may result from XII nerve injury. If the nerve is accidentally cut, primary reanastomosis should be attempted first. If primary anastomosis fails, then a greater auricular nerve graft should be done.

Postoperative shoulder pain and weakness are typically the results of XI nerve injury. This may result in significant disability. First bite syndrome is a complication that occurs when the sympathetic supply to the ipsilateral parotid gland is severed. The resultant parotid gland has an unopposed parasympathetic supply with extensive salivation. To date, no successful treatment for this syndrome exists [24].

In this work, none of these complications occurred, with only 2 patients (10.5%) with tumors Shamblin type II suffering XII nerve paralysis as a result of resection of a segment of the nerve involved within the tumor.

In cases of bilateral CBP resection, loss of the bilateral lowering blood pressure nerves occurs resulting in postoperative labile blood pressure, a condition which is difficult to control medically. Drugs targeting excess sympathetic tone, such as sodium nitroprusside, are used to control hypertension in the early postoperative period. Controlling hypertension postoperatively in these patients is critical, especially for those with vascular repair or graft. Therefore, concurrent excision of bilateral CBPs should be avoided, opting instead for two staggered surgeries that might promote compensation provided by the aortic receptors [25].

In this study, none of our patients suffered bilateral disease or were confirmed to be of the familial types. Most patients (13; 68.4%) were right-sided; however, this peculiar side does not seem to carry any significance for outcome.

Recent advances in cytogenetics identified the association of hereditary CBPs with a germline mutation. These are located in the PLG1-PLG4 genes which encode various subunits of the succinate-ubiquinone oxidoreductase gene (SDH), an enzyme involved in the mitochondrial respiratory chain complex II. These genes include SDHD (on 11q23), SDHC (on 1q21), and SDHB (on 1p36). This test helps to rule out familial types especially for paraganglioma patients <40 years of age. SDH carriers also require follow-up because of the risk of multiple tumors [26].

## 5. Conclusion

The majority of CBPs are benign. Proper diagnosis and optimum preoperative evaluation are required. Treatment involves complete R0 resection. Malignant CBPs are aggressive and are treated with surgical resection plus LN dissection followed by RT/CTH.

## Figures and Tables

**Table 1:** Incidence of symptoms associated with carotid body paragangliomas.

Pulsating painless neck lump	19 cases (100%)
Hoarseness (recurrent laryngeal nerve)	7 cases (36.8%)
Odynophagia (IX nerve)	5 cases (26.3%)
Lymphadenopathy (malignant)	3 cases (15.8%)
Dysarthria and saliva dripping (XII nerve)	1 case (5.3%)

**Table 2:** Treatment modalities for carotid body paragangliomas.

Surgery	19 cases (100%) transcervical approach
RT	2 cases (10.5%) metastatic to bone
CTH	1 case (5.3%) metastatic to lung
RT/CTH	1 case (5.3%) metastatic to lung and bone

## Data Availability

The datasets generated and analyzed during this study are available from the corresponding author upon reasonable request.
